# Characterizing the Tumor Microenvironment and Its Prognostic Impact in Breast Cancer

**DOI:** 10.3390/cells13181518

**Published:** 2024-09-10

**Authors:** Wenjuan Zhang, Alex Lee, Amit K. Tiwari, Mary Qu Yang

**Affiliations:** 1 MidSouth Bioinformatics Center and Joint Bioinformatics Graduate Program, University of Arkansas for Medical Sciences, Little Rock, AR 72204, USA; 2 Biology Department, University of Arkansas at Little Rock, Little Rock, AR 72204, USA; 3 Department of Pharmaceutical Sciences, College of Pharmacy, University of Arkansas for Medical Sciences, Little Rock, AR 72205, USA

**Keywords:** tumor immune infiltration, tumor microenvironment, immunosuppression, cell communication

## Abstract

The tumor microenvironment (TME) is crucial in cancer development and therapeutic response. Immunotherapy is increasingly recognized as a critical component of cancer treatment. While immunotherapies have shown efficacy in various cancers, including breast cancer, patient responses vary widely. Some patients receive significant benefits, while others experience minimal or no improvement. This disparity underscores the complexity and diversity of the immune system. In this study, we investigated the immune landscape and cell–cell communication within the TME of breast cancer through integrated analysis of bulk and single-cell RNA sequencing data. We established profiles of tumor immune infiltration that span across a broad spectrum of adaptive and innate immune cells. Our clustering analysis of immune infiltration identified three distinct patient groups: high T cell abundance, moderate infiltration, and low infiltration. Patients with low immune infiltration exhibited the poorest survival rates, while those in the moderate infiltration group showed better outcomes than those with high T cell abundance. Moreover, the high cell abundance group was associated with a greater tumor burden and higher rates of TP53 mutations, whereas the moderate infiltration group was characterized by a lower tumor burden and elevated PIK3CA mutations. Analysis of an independent single-cell RNA-seq breast cancer dataset confirmed the presence of similar infiltration patterns. Further investigation into ligand–receptor interactions within the TME unveiled significant variations in cell–cell communication patterns among these groups. Notably, we found that the signaling pathways SPP1 and EGF were exclusively active in the low immune infiltration group, suggesting their involvement in immune suppression. This work comprehensively characterizes the composition and dynamic interplay in the breast cancer TME. Our findings reveal associations between the extent of immune infiltration and clinical outcomes, providing valuable prognostic information for patient stratification. The unique mutations and signaling pathways associated with different patient groups offer insights into the mechanisms underlying diverse tumor immune infiltration and the formation of an immunosuppressive tumor microenvironment.

## 1. Introduction

Despite advancements in early diagnosis and treatment, breast cancer remains a leading cause of cancer-related death among women [1]. The complexity and heterogeneity of breast cancer continue to pose significant challenges in achieving effective and durable responses for many patients [2]. Immunotherapy is emerging as a critical component of breast cancer treatment [3,4]. Initially, breast cancer was not extensively investigated for its susceptibility to immunotherapy, as it was considered to have low immunogenicity [4]. However, with growing evidence of the immune system’s influence, various immunotherapeutic strategies are now being explored, including CAR-T and CAR-NK cell therapies, cancer vaccines, and cytokine inhibitors [5]. Immunotherapy leverages the body’s immune response to specifically target cancer cells, potentially reducing toxicity compared to traditional treatments and offering the possibility of durable responses that may lead to long-term remission [6]. Additionally, immunotherapy holds promise for treating breast cancer subtypes that are less responsive to conventional therapies.

Immune infiltration within the tumor microenvironment (TME) significantly influences the outcome of immunotherapy and other anti-tumor treatments. Immune cells can either bolster or restrain tumor growth based on their composition and functional states within the TME. High levels of tumor-infiltrating lymphocytes (TILs) are often associated with better responses to immune checkpoint inhibitors and improved patient outcomes [7,8]. Conversely, a TME dominated by immunosuppressive cells, such as regulatory T cells (Tregs) or myeloid-derived suppressor cells (MDSCs), can hinder the effectiveness of immune therapies, leading to poorer clinical outcomes [9]. For instance, triple-negative breast cancer (TNBC) patients with active lymphocyte-mediated immunity tend to have better prognoses [10]. Additionally, immune cells have been implicated in developing resistance mechanisms to immunotherapy in breast cancer, which hampers the establishment of durable responses and leads to disease progression [11]. Thus, a comprehensive characterization of the immune landscape is essential for improving breast cancer treatment strategies and enhancing the efficacy of immunotherapy.

Beyond a diverse array of immune cells, the TME contains other critical components, including stromal cells such as cancer-associated fibroblasts (CAFs) and endothelial cells, as well as non-cellular components of the extracellular matrix. These components interact dynamically, influencing tumor progression and the immune response [12,13]. However, despite their critical role, the characteristics and precise mechanisms of cell communication within the TME and their contribution to immune response remain incompletely understood.

In this study, we devised a method that integrates bulk and single-cell RNA sequencing data from multiple breast cancer patient cohorts to characterize the immune infiltration landscape and infer cell communication networks across diverse cell types within the tumor microenvironment (TME). Our analysis revealed three distinct breast cancer patient groups, each with unique immune infiltration profiles and clinical outcomes. Combining single-cell RNA sequencing data with ligand–receptor interaction analysis in the TME, we identified key signaling pathways involved in immune activation and suppression. Our findings provide valuable insights into the mechanisms governing the pro-tumor and anti-tumor roles of the immune system, potentially unveiling novel therapeutic targets aimed at modulating immune responses. Understanding TME-related characteristics associated with favorable or poor responses to therapy could facilitate patient stratification and inform therapeutic decisions.

## 2. Materials and Methods

### 2.1. Breast Cancer Patient Cohorts

We gathered comprehensive data for our study from multiple sources. We downloaded RNA sequencing and clinical data for 1108 breast cancer patients from breast invasive carcinoma TCGA firehose legacy. We also obtained independent breast cancer data (GSE22219) from the United Kingdom (UK) via the Gene Expression Omnibus (GEO), containing transcriptomic and clinical information for 216 breast cancer patients [14].

Moreover, we obtained single-cell RNA sequencing data (GSE176078) from GEO, comprising 26 breast cancer patients [15]. This dataset includes the expression of 29,733 genes across 100,064 cells, encompassing three breast cancer subtypes: ER+ (38,241 cells), HER2+ (19,311 cells), and TNBC (42,512 cells). It comprises diverse cell populations, listed as follows: 3206 B cells, 6573 cancer-associated fibroblasts (CAFs), 24,489 epithelial cells, 7605 endothelial cells, 9675 myeloid cells, 4355 breast epithelial cells, 3524 plasmablasts, 5423 perivascular-like (PVL) cells, and 35,214 T cells.

### 2.2. Assessment of Tumor Immune Infiltration

We obtained gene signatures for each immune type from the study by Bindea et al. [16]. These gene markers were established utilizing three gene expression datasets [17,18,19] generated from sorted immune cell populations. Angiogenesis gene signatures were extracted from Masiero et al. [20], while a signature for antigen presentation was created using genes specifically involved in the processing and presentation of antigens on Major Histocompatibility Complex (MHC) molecules [21].

We assessed the infiltration levels of immune cell types and activity levels of angiogenesis and antigen presentation using single sample Gene Set Enrichment Analysis (ssGSEA) [22], implemented through the R package GSVA [23]. The ssGSEA score reflects how a specific gene list is coordinately up- or downregulated relative to the remaining genes in the genome.

### 2.3. Computing Tumor Mutation Burden

We employed the R package maftools [24] to calculate the Tumor Mutation Burden (TMB) in TCGA breast cancer patients. The TCGA mutation file, downloaded from cBioPortal [25], was imported into R using the read.maf function. Subsequently, TMB was calculated using the tmb function with a capture size of 38.

### 2.4. Copy Number Variation Identification

We utilized GISTIS2 [26], a web tool designed to identify regions with significant deletions and amplifications, for copy number variation (CNV) analysis. The TCGA segmented data obtained from cBioPortal [25] was imported into GISTIS2, with hg19 as the reference genome. The output file (named All thresholded by genes.txt) was used to determine deletions and amplifications, where negative values indicate deletions and positive values indicate amplifications. We then calculated the number of amplifications and deletions for three groups of patients, categorized based on immune infiltration levels. The CNV counts were normalized by dividing by the total number of genes.

### 2.5. Survival Analysis

The association between clinical outcomes and patient groups was evaluated using the Cox proportional hazards regression model, implemented via the R package survival. Age was included as a covariate in the regression model. The log-rank test was adopted to determine whether patient survival significantly differed among the distinct groups.

### 2.6. Single-Cell RNA Sequencing Data Process

We filtered out cells with more than 10% mitochondrial gene expression and retained genes expressed in over 10 cells. The R package Seurat 5.0.3 [27] was used for data normalization, dimensionality reduction, cell clustering, and visualization. First, the expression matrix was loaded into R to create a Seurat object. After normalization, we extracted the top 3000 most variable genes and performed principal component analysis (PCA). Based on the top 10 principal components, cell clusters were identified using the FindClusters function with a resolution of 0.5. Finally, we visualized the cell clusters using uniform manifold approximation and projection (UMAP).

### 2.7. Cell Communication Network Inference

We employed CellChat [28] (2.1.1) to analyze intercellular communication in the TME. CellChat models the probability of cell communication by integrating gene expression data from scRNA-seq with a ligand–receptor interaction database. Active ligand and receptor interactions were identified based on differentially overexpressed signaling genes (*p* < 0.05) in each cell type. The communication probability of a signaling pathway was computed by summarizing the probabilities of its associated ligand–receptor pairs. The contributions of specific ligand–receptor pairs in a pathway were assessed using the netAnalysis_contribution function. Significant interactions between two cell clusters were determined using a permutation test by randomly shuffling the labels of cell types. The interactions with *p* < 0.05 are considered significant. The netVisual_heatmap function was employed to visualize the detailed differential number of cell–cell communications and their strengths between different immune infiltration groups in the TME.

## 3. Results

### 3.1. Immune-Infiltration Decomposition in Breast Cancer

To characterize the tumor microenvironment (TME) in breast cancer, we first investigated tumor immune infiltration, encompassing a spectrum of 24 adaptive and innate immune cell types using RNA sequencing data from 1108 TCGA breast cancer patients. A gene-signature-based approach was adopted in the analysis, with these gene signatures derived from gene expression datasets of sorted immune cell populations [16]. The infiltration levels of each immune cell type in the individual tumor samples were quantified using ssGSEA, which compares the over-expression level of the list of gene signatures to other genes in the genome [29]. Additionally, we measured the activity levels of two other components: antigen-presenting machinery (APM) and angiogenesis. APM is associated with immune cell infiltration, and its down-regulation or loss is a common immune escape mechanism in cancer [30].

**Figure 1:**
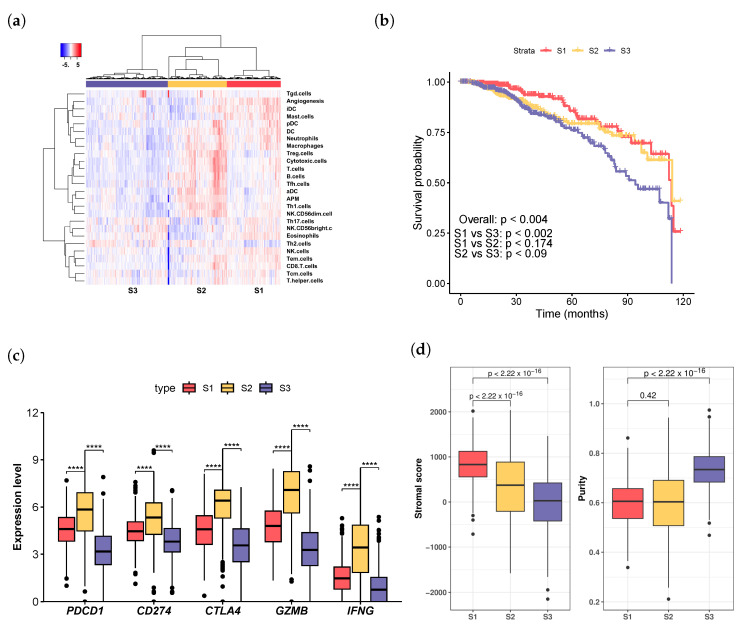
Analysis of breast cancer patient subgroups derived from immune infiltration profiles. (**a**) Hierarchical clustering based on immune infiltration levels revealed patient groups in the TCGA dataset: high T cell abundance (S2), moderate infiltration (S1), and low infiltration (S3). (**b**) The moderate infiltration group (S1) showed the best survival rate, while the low infiltration group (S3) had the poorest survival. (**c**) Immune checkpoint genes *PDCD1*, *CD274*, and *CTLA4*, as well as genes associated with T cell response, *GZMB*, and *IFNG*, were significantly more highly expressed in the T cell abundance group (S2) than in the other two groups (S1 and S3). **** p<0.0001. (**d**) The moderate infiltration group (S1) demonstrated the highest stromal score, while the low infiltration group (S3) had the highest tumor purity. The *p*-values were calculated using the Mann–Whitney U test.

Hierarchical clustering analysis, which leveraged immune infiltration levels and activity scores of APM and angiogenesis, revealed three distinct patient groups: high T cell abundance (S2), moderate infiltration (S1), and low infiltration (S3) (Figure 1a). Patients in the S3 group exhibited overall low levels of immune infiltration, while those in both S1 and S2 demonstrated significantly higher infiltration levels with distinct patterns (Figure 1a). Specifically, the S2 group was characterized by elevated T cell infiltration, whereas patients in S1 displayed intermediate levels of T cell infiltration (Figure 1a).

Survival analysis using Cox proportional hazard regression models revealed significant disparities in survival rates among the three patient groups (*p* < 0.004, Figure 1b). Patients in the moderate infiltration (S1) group demonstrated the most favorable survival rates, whereas those in the low infiltration (S3) group exhibited the poorest survival outcomes. Notably, the survival curves of the moderate infiltration (S1) cohort and the high T cell abundance (S2) cohort diverged distinctly around the 5-year mark but converged thereafter (Figure 1b). Conversely, the survival curves of patients in the high T cell abundance (S2) group and the low infiltration (S3) group showed similar trajectories before five years, diverging after that point (Figure 1b). This phenomenon may reflect the dynamic evolution and complex interactions within the TME during tumor progression.

We investigated the potential associations between various clinical factors, including age, tumor stage, surgical procedures, and cancer subtypes, with distinct immune infiltration groups. The results of the Kruskal–Wallis test indicated a significant difference in age among the three groups (*p* < 4.4 × $10^{-4}$; Supplementary Figure S1a,b). The low infiltration group (S3) exhibited the highest median age of 60 years, while the high T cell abundance group (S2) had the lowest median age of 56 years (Supplementary Figure S1a).

By contrast, no significant differences were found in tumor stage (Kruskal–Wallis test, *p* > 0.66; (Supplementary Figure S1c,d) or among histological diagnosis categories, which primarily included ductal carcinoma and infiltrating lobular carcinoma (Kruskal–Wallis test, *p* > 0.36; Supplementary Figure S1e,f). Utilizing immunohistochemistry (IHC) results, breast cancer patients were classified into three subtypes: ER+, HER2+, and TNBC [31]. Specifically, ER+ samples were defined as estrogen receptor (ER)-positive and progesterone receptor (PR)-negative; HER2+ samples were characterized by HER2 positivity regardless of ER or PR status, while TNBC samples were identified as negative for all three receptors: ER, PR, and HER2. The Kruskal–Wallis test revealed no significant associations between the subtypes and the immune infiltration groups (Kruskal–Wallis test, *p* > 0.8; Supplementary Figure S1g,h).

We examined the range of surgical procedures, including modified radical mastectomy, simple mastectomy, and lumpectomy, finding no significant association between these procedures and immune infiltration levels (Kruskal–Wallis test, *p* > 0.29; Supplementary Figure S1i,j). Analyses of tissue sampling methods (primarily core needle biopsy, tumor resection, and fine-needle aspiration biopsy) also showed no significant relationships with the immune infiltration groups (Kruskal–Wallis test, *p* > 0.32; see Supplementary Figure S1k,l).

These findings suggested that, while age was significantly associated with immune infiltration patterns, other clinical factors such as tumor stage, histological diagnosis, breast cancer subtypes, surgical procedures, and tissue sampling methods did not significantly impact classification into the identified immune infiltration groups. Therefore, the clinical outcomes observed in each infiltration group were most likely primarily attributed to the immune response.

We analyzed the expression of immune checkpoint molecules *PDCD1*, *CD274*, and *CTLA4*, which encode the immunotherapeutic targets PD-1, PD-L1, and CTLA-4, respectively. Inhibition of PD-1, PD-L1, or CTLA-4 can relieve the brakes on the immune system, allowing T cells to recognize and attack cancer cells effectively. Additionally, we examined the expression of two effector molecules associated with T cell responses, *GZMB* (Granzyme B) and *IFNG* (Interferon Gamma). We observed significantly elevated expression of these five genes within the high T cell abundance (S2) group compared to the other two groups (Figure 1c, Mann–Whitney U test). Taken together, the markedly lower 5-year survival rate observed in the high T cell abundance (S2) group compared to the moderated infiltration group (S1) (*p* < 0.026) suggests that the pro-survival impact of high levels of T cell infiltration and its effector molecules in S2 was counteracted by immune inhibitors and factors such as exhaustion.

Moreover, we evaluated the stromal fraction in the tumor samples utilizing the gene-expression-profile-based ESTIMATE (Estimation of Stromal and Immune cells in Malignant Tumours using Expression data) method [32] and then studied its association with the groups (Figure 1d). The low immune infiltration group (S3) showed the lowest stromal scores (*p* < 2 × $10^{-16}$, ANOVA), while the moderate infiltration (S1) group displayed higher stromal scores (*p* < 2.2 × $10^{-16}$, Mann–Whitney U test) than the high T cell abundance group (S2). Tumor purity was determined using the gene-expression-profile-based PUREE algorithm [33], confirming that the low immune infiltration (S3) group displayed the highest tumor purity (Figure 1d).

To further assess the influence of stromal content on patient survival, we constructed a univariate Cox regression model. The resulting hazard ratio of 1 (95% CI: 0.998, 1) indicates that stromal content exerts a negligible effect on the hazard of death. The corresponding *p*-value of 0.962 reinforces this finding, suggesting no significant relationship between stromal fraction and patient survival. Therefore, the favorable patient outcomes observed in the moderate infiltration group (S1) appear to be primarily due to immune infiltration rather than stromal content.

To validate that the three tumor infiltration groups are not unique in the TCGA breast cancer cohort, we examined an independent patient cohort (GSE22219) containing 216 breast cancer patients [14]. This analysis revealed similar immune infiltration groups within the dataset (Supplementary Figure S2a). Patients with low immune infiltration had the poorest survival rate, whereas those with high T cell infiltration had lower survival rates compared to patients in the moderate infiltration (S1) group (Supplementary Figure S2b).

### 3.2. Exploring Infiltration Patterns across Patient Groups

**Figure 2:**
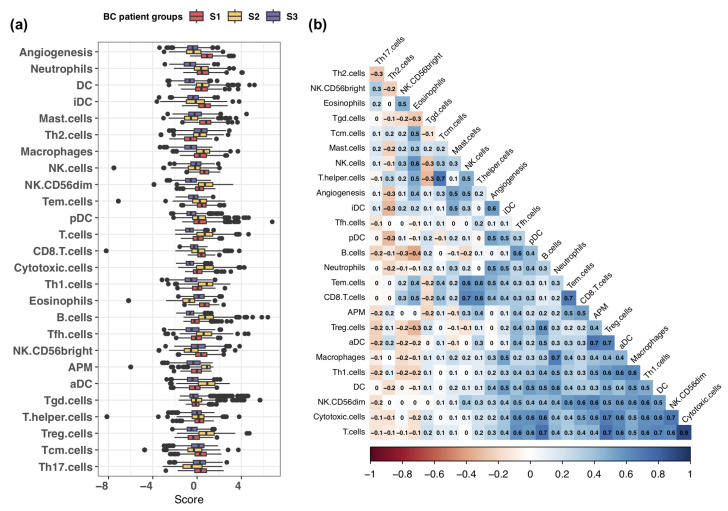
Analysis of immune infiltration and activity scores. (**a**) Comparison of infiltration levels of 24 innate and adaptive immune cells, along with activity scores for angiogenesis and antigen-presenting machinery (APM), across the three patient groups. The immune cells were ranked based on differences between the moderate infiltration (S1) and low infiltration (S3) groups, which were associated with favorable and poor patient survival, respectively. (**b**) Pearson correlation analysis of immune cell infiltration levels and activity levels of angiogenesis and APM.

We analyzed the distribution statistics of the infiltration and activity scores across distinct patient groups. Subsequently, we ranked the immune cells based on the absolute median difference in scores between those associated with favorable survival rates (moderate infiltration, S1) and those linked to poor survival rates (low infiltration, S3) (Figure 2a). The top-ranked immune cells included neutrophils, dendritic cells (DCs), immature dendritic cells (iDCs), mast cells, T helper 2 (Th2) cells, and natural killer (NK) cells.

Neutrophils play dual roles in tumor progression, initially inhibiting tumor development by promoting cytotoxic T cell proliferation [34] and producing various cytokines and growth factors [35,36]. However, as tumors progress, these functions become ineffective, and neutrophils acquire pro-tumor properties [37,38,39]. DCs, a specialized type of antigen-presenting cells, play a crucial role in initiating and regulating innate and adaptive immune responses [40]. Tumor-infiltrating DCs have been associated with improved survival and reduced recurrence rates in breast cancer patients [41]. Mast cells, innate immune cells derived from myeloid stem cells, exhibit either pro-tumorigenic or anti-tumorigenic functions depending on factors such as cancer type, tumor stage, and their localization within the tumor microenvironment [42,43]. Natural killer (NK) cells, a subset of cytotoxic innate lymphoid cells, secrete inflammatory cytokines and chemokines and limit tumor growth by lysing transformed or infected cells [44].

We found that the infiltration levels of DCs and neutrophils were higher in the moderate infiltration (S1) and high T cell abundance (S2) groups compared to the low infiltration group (S3). Additionally, mast cells and natural killer (NK) cells exhibited higher infiltration levels in the moderate infiltration (S1) group than in the other two groups. By contrast, Th2 cell infiltration was significantly lower in the moderate infiltration (S1) group than in the other two groups. High infiltration of Th2 cells has been associated with aggressive features of breast cancer [45].

Interestingly, we observed that the moderate infiltration (S1) group displayed the highest angiogenesis scores, even though angiogenesis is well known for facilitating tumor growth and metastasis [46,47]. To further investigate this observation, we calculated Pearson correlation coefficients (PCCs) to assess the relationships between angiogenesis activity and immune cell infiltration. The immune cells exhibited strong positive correlations, including dendritic cells (DCs) (PCC = 0.41), neutrophils (PCC = 0.51), mast cells (PCC = 0.52), and natural killer (NK) cells (PCC = 0.48). By contrast, Th2 cells exhibited a negative correlation (PCC = −0.27) (Figure 2b, Supplementary Table S1). Thus, the active angiogenesis in the moderate infiltration (S1) group may have arisen from the higher levels of infiltration of these immune cells.

### 3.3. Relation of Genetic Mutations with Immune Infiltration

One significant consequence of genetic mutations in cancer is the modulation of immune infiltration within the tumor microenvironment. Certain mutations can promote the recruitment of immune cells, such as T cells, natural killer cells, and macrophages, to the tumor site [48,49]. Conversely, other mutations may facilitate immune evasion mechanisms, thereby reducing the immune response against the tumor [50].

**Figure 3:**
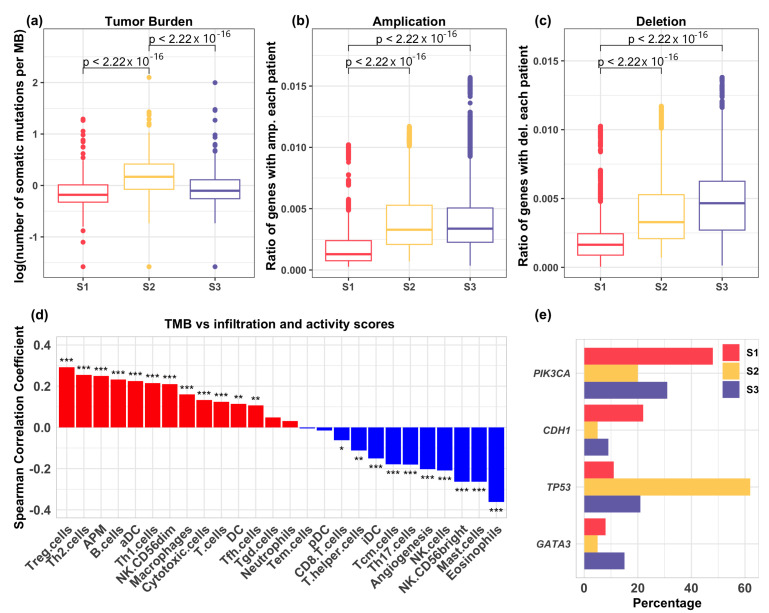
(**a**) Comparison of tumor burden across the three immune infiltration groups. Mann–Whitney tests revealed a significantly higher tumor burden in the high T cell abundance (S2) group compared to the moderate infiltration (S1) and the low infiltration (S3) groups. (**b**) Analysis of amplification regions in the genomes among patients in distinct groups. The moderate infiltration group (S1) exhibited significantly lower rates of amplification compared to the other groups, as determined by Mann–Whitney tests. (**c**) Analysis of deletion regions in the genomes among patients in distinct groups. The moderate infiltration group (S1) showed significantly lower deletion rates than the other two groups, as determined by Mann–Whitney tests. (**d**) Spearman correlation analysis between tumor burden and the infiltration of each immune cell type, as well as angiogenesis and APM activity scores. * p<0.05, ** p<0.01, *** p<0.001. (**e**) Identification of the top mutated genes in the three immune infiltration groups. *TP53* was highly mutated in the high T cell abundance (S2) group, whereas *PIK3CA* was predominantly mutated in the moderate infiltration group (S1).

We assessed the tumor burden, defined as the total number of somatic mutations per megabase pair, and its association with the groups. We found that patients in the moderate infiltration group (S1) harbored the lowest somatic mutation rates, while those in the high T cell abundance group (S2) exhibited the highest mutation burden (Figure 3a). Copy number variation (CNV) analysis indicated that the moderate infiltration (S1) group had the lowest amplification and deletion rates, while the high T cell abundance group (S2) had lower deletion rates compared to the low infiltration group (S3) (Figure 3b,c).

We performed Spearman correlation analysis to assess the relationship between mutation burden and tumor immune infiltration. Unlike mast cells, NK cells, and angiogenesis, the infiltration levels of regulatory T (Treg) and Th2 cells were significantly positively correlated with mutation burden (Figure 3d). These correlations may suggest an immunosuppressive microenvironment enriched with Treg and/or Th2 cells, potentially facilitating immune evasion, despite the presence of immunogenic mutations. Additionally, the activity score of antigen presentation machinery (APM) was positively correlated with mutation load, indicating that tumor immune infiltration can upregulate the expression of APM genes through paracrine signaling and mRNA production by infiltrating cells [51].

We further investigated mutations in individual genes and found that the mutation rates of *PIK3CA*, *TP53*, *CDH1*, and *GATA3* significantly differed across the patient groups (Figure 3e). *PIK3CA*, which encodes the catalytic subunit of PI3K, can lead to the constitutive activation of the PI3K pathway, crucial for regulating cellular processes such as metabolism, proliferation, and survival [52,53]. Reported mutation rates for *PIK3CA* in breast cancer range from 18% to 40% [54,55,56]. Here, we found a notably high mutation rate in the moderate infiltration (S1) group (48%), compared to 20% in the high T cell abundance (S2) group and 31% in the low infiltration (S3) group. Conversely, *TP53* mutations were relatively low in the moderate infiltration (S1) group (11%) compared to 62% in the high T cell abundance (S2) group and 21% in the low infiltration (S3) group (Figure 3e). *TP53* encodes the transcription factor P53, which is pivotal for regulating genes involved in cellular processes such as cell cycle arrest, apoptosis, metabolism, and DNA repair [57]. The reported mutation rate of *TP53* in breast cancer is approximately 30% [58]. Notably, the loss of P53 function has been linked to immunosuppressive mechanisms through the upregulation of PD-1 and PD-L1, which act as critical immune checkpoints and play a central role in T cell exhaustion [59]. Somatic mutations in *CDH1* are found in around 50% of lobular breast cancers [60,61], while *GATA3* mutations occur in approximately 10% of breast cancers [62]. Tumors in the moderate infiltration (S1) group show higher mutation rates of *CDH1* but lower rates of *GATA3*. *GATA3* is a transcription factor involved in regulating Th2 and regulatory T (Treg) cells in immune regulation and tolerance [63,64].

Collectively, tumors in the moderate infiltration (S1) group were characterized by high mutation rates of *PIK3CA* and *CDH1*, while those in the high T cell abundance (S2) group exhibited elevated mutations in *TP53*. By contrast, the low infiltration group (S3) showed a higher mutation rate in *GATA3* compared to the other groups. These mutation patterns may be associated with distinct immune infiltration profiles; however, causal relationships require further investigation.

### 3.4. Breast Cancer Patient Clusters Revealed by scRNA-seq Data

**Figure 4:**
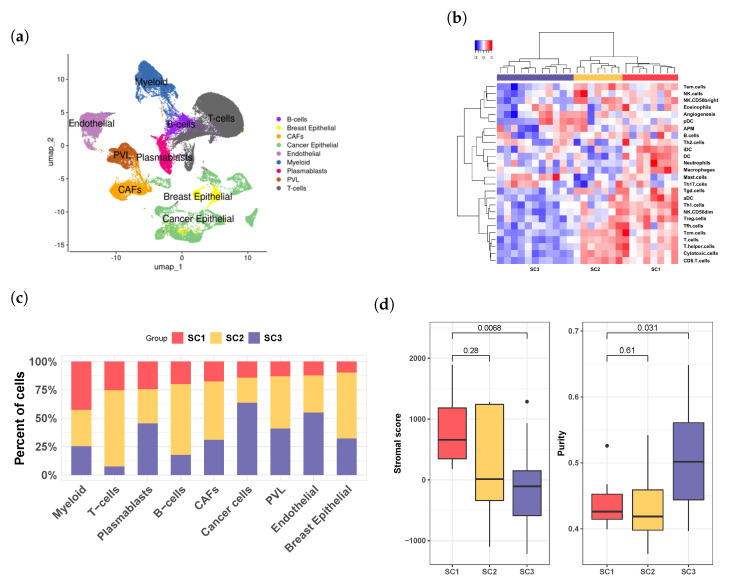
Analysis of single-cell RNA sequencing data from breast cancer patients. (**a**) Visualization of major cell types in the tumor microenvironment (TME) using UMAP. (**b**) Hierarchical clustering analysis of the pseudo-bulk expression data revealed three distinct infiltration groups: SC1, SC2, and SC3. These groups, represented by red, yellow, and purple sidebars, respectively, closely mirror the groups identified in the TCGA bulk RNA sequencing data (S1, S2, and S3). (**c**) Distribution of the three infiltration groups across different cell populations. (**d**) Evaluation of stromal score and tumor purity across the three groups, demonstrating trends in SC1, SC2, and SC3 similar to those observed in S1, S2, and S3 from the TCGA dataset.

To characterize cell–cell communication within the tumor microenvironment (TME), we expanded our analysis to include single-cell RNA sequencing data (GSE176078) from 26 breast cancer patients, comprising 11 ER+, 5 HER2+, and 10 TNBC cases. After excluding low-quality genes and cells, our dataset included 24,412 genes and 84,482 cells for further analysis. Cell types were visualized using the uniform manifold approximation and projection (UMAP) method (Figure 4a).

We first derived pseudo-bulk data from this dataset by normalizing the average expression counts across all cells for each patient. We then calculated tumor infiltration levels of immune cells using a signature-based method similar to that employed in the TCGA data analysis. Hierarchical analysis of immune infiltration levels revealed three patient groups, referred to as SC1, SC2, and SC3. SC1 and SC2 exhibited high immune infiltration, while SC3 demonstrated low infiltration levels (Figure 4b). Compared to SC1, SC2 had a higher proportion of T cells (Figure 4c). Furthermore, the SC3 group had significantly lower stromal scores (Figure 4d; *p* < 0.0068, Mann–Whitney U test) and higher tumor purity than SC1 (*p* < 0.031, Mann–Whitney U test). Collectively, the characteristics of the SC1, SC2, and SC3 groups identified in the scRNA-seq data illustrated similar immune infiltration patterns (Figure 1a vs. Figure 4b), as well as stromal scores and tumor purity (Figure 1d vs. Figure 4d), comparable to those of the moderate infiltration (S1), high T cell abundance (S2), and low infiltration (S3) groups in the TCGA and UK breast cancer datasets, suggesting their similarity in the TME.

### 3.5. Deciphering Cellular Interactions in Tumor Microenviroment

Cell–cell interactions and communication are essential for cellular functions and immune responses. In this study, we employed CellChat [28] to investigate intercellular communications in the tumor microenvironment (TME), focusing on patients in the SC1 and SC3 groups. These two groups demonstrated significant differences in immune infiltration and other characteristics of the TME (Figure 4b,d), as well as potential clinical outcomes. Group SC1 is characterized as immune-active, displaying robust immune responses, whereas group SC3 is immune-suppressed, exhibiting reduced immune activity.

**Figure 5:**
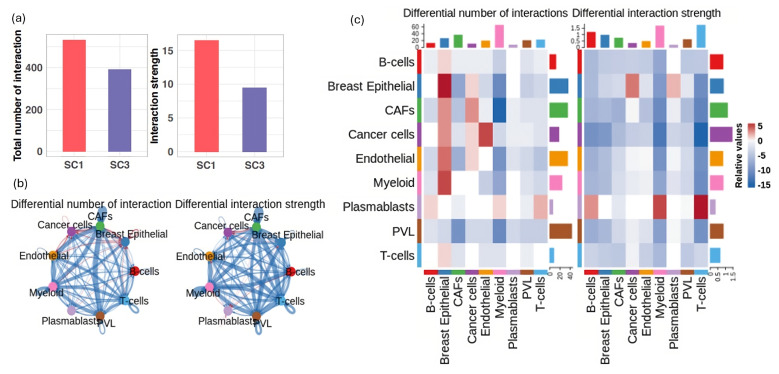
Overview of the cell–cell communications in the immune-active (SC1) and immune-suppressed (SC3) groups. (**a**) The number of interactions (**left**) and interaction strength (**right**) within each group. (**b**) Changes in the number (**left**) and strength (**right**) of cell–cell communications between the immune-active and -suppressed groups. Blue arrows indicate a decrease in the number or strength of interactions in the immune-suppressed group compared to the immune-active group, while red arrows indicate an increase in these metrics in the immune-suppressed group. In the weighted directed graphs, the arrows represent the signaling pathways, pointing from the signal-originating cell type (ligand-producing) to the target cell type (signal-receiving). The width of the arrows reflects the differences in the number or strength of communication interactions between the two groups. (**c**) Detailed analysis of differential cell–cell communication numbers (**left**) and communication strength (**right**) between the immune-active and -suppressed groups. Cell types are plotted on the x-axis as receivers and on the y-axis as senders of communication signals. The color gradient from red to blue indicates decreasing values in the number or strength of interactions in the immune-suppressed group relative to the immune-active group.

We evaluated the quantity and strength of intercellular interactions within the immune-active (SC1) and -suppressed (SC3) groups. Our findings revealed that both the total number and strength of cell communications were significantly lower in the immune-suppressed group compared to the immune-active group (Figure 5a). Further differential analysis demonstrated that cell interactions were prevalent in the TME of the immune-active group; however, many of these interactions were either diminished or lost in the immune-suppressed group, as depicted by the blue arrows in Figure 5b. In these weighted directed graphs, arrows indicate the direction of signaling, pointing from the ligand-producing cell type (signal sender) to the target cell type (signal receiver). The width of the arrows reflects the extent of difference in either the number (left panel of Figure 5b)or the strength (right panel of Figure 5b) of communication between the two immune response groups. Blue represents decreased or lost cellular interactions, while red indicates increased or gained cellular interactions in the immune-suppressed group (Figure 5b and Figure 5c, respectively). For instance, the strong interactions observed in the immune-active group between T cells and various other cell types—such as cancer cells, cancer-associated fibroblasts (CAFs), myeloid cells, and endothelial cells—markedly decreased in the immune-suppressed group. This reduction suggests compromised immune responses and altered TME dynamics in the patients experiencing immune suppression.

Despite the overall decline in cellular interactions, certain interactions increased within the immune-suppressed group (red color, Figure 5b,c). The interactions between plasmablasts and multiple cell types, including B cells, myeloid cells, and T cells, increased in both number and strength in the immune-suppressed group. This phenomenon likely reflects a complex adaptive response to the suppressive tumor microenvironment. These enhanced interactions may help maintain some level of immune functionality and surveillance, counteract suppressive signals, and potentially facilitate a specific type of immune response that can persist despite overall immune suppression.

### 3.6. Major Cell Communication Patterns within the TME

**Figure 6:**
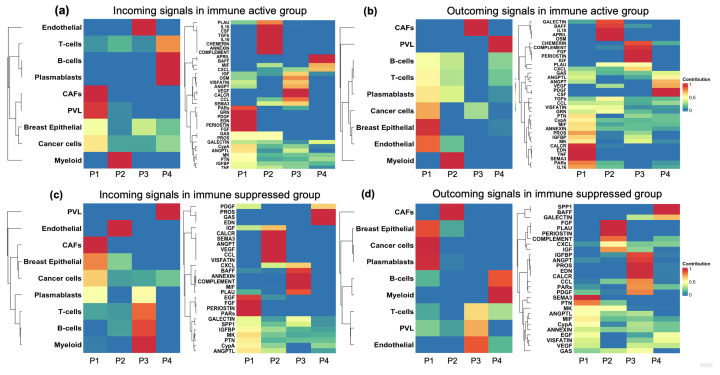
Cell–cell communication patterns coordinated by various cell types and signal pathways. This figure illustrates four communication patterns (P1, P2, P3, and P4) linking cell types and signaling pathways within the TME of the immune-active and -suppressed groups. The color gradient from red to blue indicates the probability of communication, ranging from 1 (high) to 0 (low). (**a**) Incoming signals in the immune-active group: The left panel displays signal-receiving cell types, while the right panel shows the pathways involved in these communication patterns. (**b**) Outgoing signals in the immune-active group: The left panel illustrates signal-sending cell types, with the right panel depicting the pathways involved. (**c**) Incoming signals in the immune suppressive group: The left panel presents signal-sending cell types, and the right panel shows the pathways involved in these communication patterns. (**d**) Outgoing signals in the immune-suppressed group: The left panel features signal-receiving cell types, while the right panel displays the associated pathways.

We utilized a non-negative matrix factorization method to uncover cell communication patterns and understand how cell types and signaling pathways coordinate to regulate tumor microenvironment (TME) immunity. This approach delineated comprehensive cell communication networks and identified critical signaling pathways across various cell types. We identified four communication patterns (P1, P2, P3, and P4, as shown in Figure 6) that connect cell types with pathways in both outgoing and incoming signaling in the immune-active and immune-suppressed groups (Figure 6). In this context, incoming signaling pertains to cells acting as signal receivers (left panels of Figure 6a,c), whereas outgoing signaling refers to cells functioning as signal senders (right panels of Figure 6b,d) in the intercellular communications.

Overall, the immune-active (SC1) and immune-suppressed (SC3) groups exhibited markedly distinct patterns of cell interplay, including differences in cell-type composition, the connectivity of signaling pathways, and the activation or suppression of outgoing and incoming signals. For instance, in the immune-active group, incoming T cell signaling was characterized by pattern four (P4, as shown in the left panel of Figure 6a), primarily driven by the APRIL, BAFF, MIF, and CXCL pathways (right panel of Figure 6a). By contrast, T cell incoming signaling in the immune-suppressed group was characterized by pattern three (P3, as shown in the left panel of Figure 6c), which was mainly regulated by the MIF, COMPLEMENT, ANNEXIN, BAFF, PLAU, and CXCL pathways (right panel of Figure 6c). It has been reported that PLAU plays a crucial role in Treg suppressor function [65], while APRIL is involved in inducing and sustaining T cell responses [66]. Thus, the shared signaling pathways may serve as critical targets for T cell modulation. Conversely, the unique pathways specific to each group, such as APRIL in the immune-active group and PLAU in the immune-suppressed group, may play a vital role in regulating T cell infiltration within the tumor.

### 3.7. Signaling Pathway Alterations Associated with Immune Activation and Suppression

**Figure 7:**
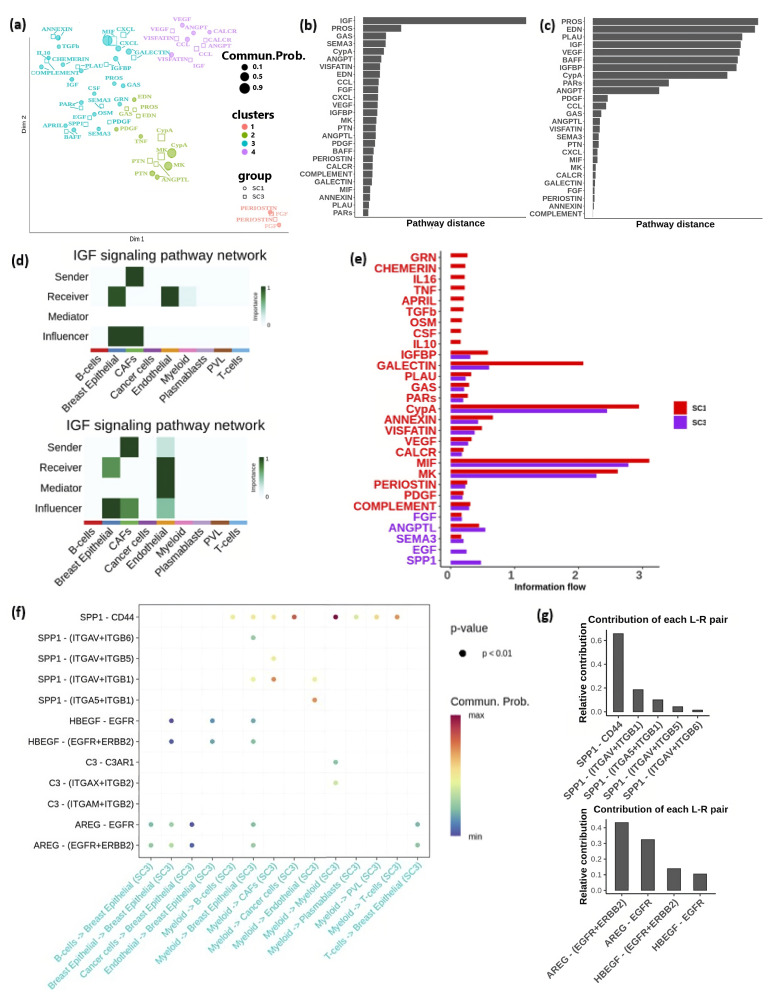
Pathways in the immune-active (SC1) and immune-suppressed (SC3) groups: (**a**) Functional similarity clusters of signaling pathways in the immune-active and immune-suppressed groups. (**b**) Overlapping pathways ranked based on functional differences. (**c**) Overlapping pathways ranked based on structural differences. (**d**) Heatmap illustrating the relative significance of each cell type derived from network centrality measures within the IGF signaling network in the immune-active group (top panel) and immune-suppressed group (bottom panel). (**e**) Ranking of signaling pathways based on their involvement in the immune-active (SC1) and immune-suppressed (SC3) groups. (**f**) Interactions of the SPP1 and EGF pathways with various cell types in the immune-active (SC1) and immune-suppressed (SC3) groups. (**g**) Top panel: Contribution of ligand–receptor pairs in the SPP1 pathway. Bottom panel: Contribution of ligand–receptor pairs in the EGF pathway.

To investigate changes in signaling pathways associated with immune variations, we developed separate intercellular communication networks for the immune-active and immune-suppressed groups. Using UMAP, we displayed the functional similarities of these pathways on a two-dimensional manifold, where each point represents an individual pathway (Figure 7a). We then applied k-means clustering to this manifold to identify patterns that categorize the pathways based on their functional relationships. Four distinct clusters were uncovered (Figure 7a). Many pathways were shared between the immune-active and immune-suppressed groups. For instance, the ANNEXIN and PAR pathways in both groups (SC1 and SC3) were closely positioned within cluster 3 (cyan, Figure 7a), indicating their involvement in both immune activation and suppression. However, they may not critically influence the immune response in breast cancer. By contrast, pathways such as IGF and PROS exhibited more pronounced functional variations across different immune groups. Specifically, the IGF pathway was identified in cluster 4 (purple, Figure 7a) within the immune-suppressed group (SC3), whereas it was located in cluster 3 (cyan, Figure 7a) in the immune-active group (SC1), suggesting that its significant functional changes contribute to distinct immune responses.

Furthermore, we calculated the Euclidean distance between common signaling pathways in the shared two-dimensional space based on their functional similarity. IGF (Insulin-like growth factor) and PROS pathways showed substantial functional changes between the two groups (Figure 7b). We also classified the signal pathways and assessed the Euclidean distance between common pathways based on network structure similarity. The results revealed notable architectural changes in pathways including PROS, EDN, PLAU, IGF, VEGF, BAFF, IGFBP, and CypA across the immune-active and -suppressed groups (Figure 7c). Conversely, pathways with small distances indicated conserved communication network structures among overlapping pathways in both immune-active and -suppressed groups.

IGF and PROS demonstrated substantial functional and structural changes in the immune-active (SC1) and immune-suppressed (SC3) groups. IGF has been implicated in promoting immunosuppression [67,68]. Network centrality analysis of the IGF signaling network revealed that endothelial cells function as mediators and influencers within the IGF pathway in the immune-suppressed group (bottom panel of Figure 7d), a role not observed in the immune-active group (top panel of Figure 7d). These mediators act as gatekeepers, controlling the flow of communication between the two cell groups. Additionally, the VEGF (Vascular Endothelial Growth Factor) signaling pathway exhibited significant structural changes between the immune-active and immune-suppressed groups (Figure 7c). Its primary function is to regulate angiogenesis.

Next, we compared the information flow of pathways, defined by overall communication probability, between the immune-active (SC1) and immune-suppressed (SC3) groups (Figure 7e). Out of the 29 pathways analyzed, 24 were found to be highly active in the immune-active group. Specifically, nine signaling pathways, including GRN, CHEMERIN, IL16, TNF, APRIL, TGFb, OSM, CSF, and IL10, were uniquely active in the immune-active cases, suggesting their roles in facilitating immune activation. By contrast, among the five pathways that were highly active in the immune-suppressed group, Epidermal Growth Factor (EGF) and Secreted Phosphoprotein 1 (SPP1) were exclusively activated in the immune-suppressed group (SC3), indicating their potential roles in promoting an immunosuppressive tumor microenvironment conducive to tumor progression. The SPP1 signaling pathway was predominantly influenced by the SPP1 ligand and the CD44 receptor (Figure 7f,g), driving most communication from myeloid cells to other cells (Figure 7f). Additionally, ligand AREG and its EGFR receptor, along with the multimeric EGFR/ERBB2 effector, are major components of the EGF pathway, facilitating signals from various cell types to breast epithelial cells (Figure 7f,g). These ligand–receptor pairs present promising targets for interventions aimed at mitigating immune suppression.

## 4. Discussion

In this study, we systematically investigated the tumor microenvironment (TME) of breast cancer, including the ER+, HER2+, and TNBC subtypes, by integrating multiple datasets that leverage information from large cohorts of patients and cell samples, along with clinical data. Our focus was on characterizing tumor immune infiltration and cell–cell communications within the TME and their connections to clinical outcomes. We summarized our analysis strategy and procedures in Supplementary Figure S3. The TME, which comprises various cell types, plays a crucial role in cancer development. Interactions among these cells and signaling molecules create a dynamic environment that can either suppress or promote tumor growth. Understanding the TME is essential for developing new therapeutic strategies, as targeting its components and interactions holds promise for improving cancer treatment and patient outcomes. Our findings offer valuable insights for patient stratification, refining treatment strategies, and elucidating the mechanisms driving the diversity of immune infiltration and intercellular communications within the TME.

We uncovered three distinct breast cancer patient groups based on immune infiltration patterns. We found that breast cancer patients with overall low immune infiltration have poor survival rates. Several studies have shown that low immune infiltration correlates with reduced overall survival and disease-free survival across various cancer types, including lung and colorectal cancers, primarily due to the lack of an effective anti-tumor immune response [69,70]. Without sufficient immune cells, the body’s ability to recognize and attack cancer cells is significantly compromised [71]. Moreover, tumors characterized by low immune infiltration often exhibit an immunosuppressive microenvironment, which can further hinder the recruitment of anti-tumor immune cells to tumor sites [72]. Clinical data also suggested that patients with low tumor immune infiltration were less likely to respond to immunotherapies [73,74].

On the other hand, we observed that patients with high T cell abundance had lower survival rates compared to those in the moderate immune infiltration group. Although high levels of cytotoxic T cells typically correlate with better prognosis due to their role in attacking cancer cells, exhausted T cells can lead to worse clinical outcomes [75]. Exhausted T cells express high levels of inhibitory receptors such as PD-1 [76], indicating chronic antigen exposure and impaired effector functions, which are associated with poor responses to therapies and reduced survival rates. Our study found increased expression levels of genes encoding PD-1, PD-L1, and CTLA-4 in the high T cell abundance group. Blocking PD-1, PD-L1, or CTLA-4 can reinvigorate T cells, allowing them to recognize and effectively attack cancer cells. Therefore, the potential benefits of high T cell infiltration on survival were mitigated by the presence of immune inhibitory mechanisms.

In addition, the patients in the T cell abundance group also expressed high levels of GZMB and IFNG. GZMB is a crucial serine protease secreted mainly by activated T cells and NK cells to trigger apoptosis [77]. Disruption in GZMB activity could be correlated with diminished entry, trafficking, and accumulation within the cytoplasm of target cells, such as tumor cells [78,79]. High GZMB expression can also be linked to immune suppression, occurring through various mechanisms, such as the induction of T cell exhaustion and the presence of Tregs and myeloid-derived suppressor cells (MDSCs), which can secrete inhibitory cytokines and create an immunosuppressive milieu. IFNG is a pleiotropic cytokine with diverse functions in the immune system, produced by activated T cells and NK cells in response to pathogens and other immune stimuli [80]. Within the TME, IFNG serves a complex role, concurrently orchestrating protumorigenic and antitumor immune responses. It collaborates with GZMB and perforin as a cytotoxic cytokine to trigger apoptosis in tumor cells [81,82]. Nevertheless, IFNG also fosters the synthesis of immune checkpoint inhibitory molecules and indoleamine-2,3-dioxygenase (IDO), thus facilitating additional immune-suppressive mechanisms [83,84].

A high tumor burden often exhibits elevated levels of neoantigens, which the immune system can recognize, leading to enhanced immune infiltration. We observed significantly higher levels of TMB in the T cell abundance group, which correlated with the increased infiltration of cytotoxic T cells, suggesting a more inflamed tumor microenvironment. Additionally, our results indicated that patients in the high T cell abundance group had a high rate of *TP53* mutations; the association between *TP53* mutations and TMB has been previously reported in lung cancer [85]. Missense mutations in *TP53* have been linked to increased genomic instability and higher T cell density [86,87,88], which is consistent with our findings. By contrast, patients with moderate immune infiltration exhibited a high rate of *PIK3CA* mutations. Research has shown that *PIK3CA* mutations can modulate the anti-tumor immune microenvironment [89].

Immune cells can both suppress and promote tumor growth, depending on their activation state and the signals they receive from the tumor microenvironment (TME). This dynamic creates selective pressures that drive tumors to acquire mutations, facilitating immune evasion or allowing them to co-opt immune cells to their advantage [13]. The diverse immune landscape significantly influences tumor evolution, resulting in distinct mutational profiles. For instance, pro-inflammatory cytokines, predominantly secreted by activated macrophages [90], can induce DNA damage and genomic instability [91]. Conversely, genetic mutations can affect the recruitment of immune cells to tumor sites, further influencing tumor dynamics by either promoting tumor growth or aiding immune evasion. For example, missense mutations in TP53 can attract pro-tumor myeloid cells [87], while mutated BRAF can promote a monocyte-derived dendritic cell (DC) phenotype that potentially affects antitumor T cell function in human melanoma [92]. These complex interactions between tumors and the immune system shape tumor behavior and genetic heterogeneity, underscoring the necessity of understanding these relationships to develop targeted immunotherapies tailored to specific mutational profiles.

We identified an increased frequency of *PIK3CA* mutations, a greater stromal fraction, and increased angiogenesis activity in the moderate immune infiltration (S1) group. Patients within this group exhibit favorable survival outcomes compared to other groups. However, the existing literature indicates that these factors may adversely affect patient survival under certain conditions or types of cancer. Dumont et al. [93] reviewed data from 12 studies encompassing 2,567 breast cancer patients and uncovered inconsistencies in clinical outcomes; PIK3CA mutations were associated with positive [94,95,96,97], negative [98,99,100], or no significant [101,102,103,104,105] impact survival. Their analysis indicated that the influence of *PIK3CA* mutations likely hinges on cancer subtypes and mutation sites. Other investigations [106,107] have demonstrated that HR+/HER2− patients with mutated PIK3CA experienced shorter survival times, while those harboring *PIK3CA* mutations in TNBC showed improved overall survival [107]. Regarding stromal content, research assessing the prognostic impact of stromal fraction in 16 solid tumors involving 2,732 patients revealed both positive (HR = 0.56 in pancreato-biliary type periampullary cancer and HR = 0.41 in estrogen-negative breast cancer) and negative (HR = 3.59 in intestinal type periampullary cancer) correlations between stromal content and patient survival [108]. In terms of angiogenesis, tumor angiogenesis is crucial for tumor advancement [109], as it supplies essential nutrients to tumors, facilitating their survival and growth. Evidence suggests angiogenesis can promote the development of various cancers, such as breast [110], colorectal [111], non-small-cell lung [112], and renal cell carcinoma [113]. However, current anti-angiogenic therapies are often ineffective; many cancer patients either lose their response to treatment or do not respond at all [114,115]. Therefore, we conducted univariate survival analyses on each factor. We uncovered no significant association between *PIK3CA* mutations and survival (*p* = 0.904, HR = 0.978; 95% CI: 0.678–1.410 Supplementary Figure S4), stromal content and survival (*p* = 0.96, HR = 1; 95% CI: 0.998–1), or angiogenesis and patient survival (*p* = 0.808, HR = 1.018; 95% CI: 0.876–1.184). Notably, despite heightened angiogenesis activity in the immune-active group, intercellular communication networks suggested that the VEGF signaling pathway, integral in regulating tumoral angiogenesis, mediates cellular interactions between cancer cells and endothelial cells in the immune-suppressed group. By contrast, such interactions were absent in the immune-active group (Supplementary Figure S5). Our findings suggest that the favorable survival rate in the moderate infiltration group (S1) primarily results from immune responses rather than direct influences of *PIK3CA* mutations, increased stromal content, or angiogenesis activity.

Beclin1 (BECN1), a haploinsufficient tumor suppressor, plays a crucial role in regulating autophagy and has been implicated in the tumorigenesis of various cancer types [116,117]. Deletions of BECN1 [118,119,120] often co-occur with deletions of the tumor-suppressor gene BRCA1 [121]; both genes are situated in close proximity on chromosome 17q21. Tang et al. studied the prognostic value of BECN1 and BRCA1 expression and found that decreased expression of BECN1 was associated with poor survival in HER2+ breast cancer patients; however, no significant association was detected between BRCA1 expression and survival in their study [122]. Similarly, we found that lower expression levels of BECN1 tended to correlate with poor survival, with a *p*-value of 0.082 and a HR of 0.804 in HER2+ patients. By contrast, BRCA1 expression showed no significant association with survival in this patient subtype (*p* = 0.964, HR = 1.005). Moreover, our results showed that both BECN1 and BRCA1 had elevated expression levels in the low immune infiltration group (ANOVA test, *p* < 2 × $10^{-16}$).

Interestingly, we observed a convergence of the survival curves for the T cell abundance group (S2) and the low immune infiltration group (S3) within the first five years (Figure 1b). We hypothesize that this may result from similar tumor biology or treatment responses in these groups, possibly overshadowing the differences in immune infiltration profiles during this period. For example, the presence of immune inhibitors and potential T cell exhaustion in the high T cell abundance group may impede an effective immune response against cancer cells. On the other hand, after five years, the similarity in survival curves between the moderate immune infiltration (S1) and T cell abundance (S2) groups might indicate evolving functional roles of certain immune cell types and other factors in the tumor microenvironment. Patients in the moderate infiltration group (S1) exhibited higher levels of mast cell infiltration compared to those in the T cell abundance (S2) group. Mast cells can exert either anti-tumor or pro-tumor effects, depending on factors such as tumor stage and location. Another potential factor is the influence of genetic mutations and the dynamic changes in other prognostic indicators. For instance, although we found no significant initial association between *PIK3CA* mutations and survival, around the five-year mark, we noticed that these mutations began to adversely affect survival outcomes (Supplementary Figure S4). This effect may be more pronounced, given that the T cell abundance group has a relatively lower PIK3CA mutation ratio compared to the moderate infiltration group. Further investigation is warranted to better understand this phenomenon, which could lead to improved treatment strategies for breast cancer patients.

Additionally, we analyzed variations in several clinical parameters across the immune infiltration groups. These parameters included age, tumor stage (I, II, III, and IV), histological diagnosis (primarily infiltrating ductal carcinoma and lobular carcinoma), subtypes (ER+, HER2+, and TNBC), surgical methods (mainly lumpectomy, modified radical mastectomy, and simple mastectomy), and tissue sampling methods (primarily core needle biopsy, tumor resection, and fine-needle aspiration biopsy). We found that only age displayed significant differences among the distinct infiltration groups (Results; Supplementary Figure S1), suggesting that levels of immune infiltration are likely a dominant factor associated with the observed clinical outcomes in each group. Breast cancer treatments typically encompass surgery, chemotherapy, radiotherapy, hormone therapy, targeted therapy, and immune therapy; surgical interventions are often combined with other treatments, such as radiotherapy and chemotherapy [123], primarily depending on the cancer’s stage, grade, and subtype [123]. However, detailed information about therapeutic regimens, aside from initial surgical types, is lacking. This limitation restricts our ability to effectively evaluate the impacts of these regimens on tumor immunity and patient survival.

Cell–cell communication in the TME is pivotal in driving cancer progression and resistance to therapies. This communication primarily occurs through ligand–receptor interactions, where various signaling molecules such as growth factors, cytokines, and chemokines bind to specific receptors on target cells. Our study focused on analyzing these interactions to uncover unique signaling pathways that regulate cell–cell crosstalk among breast cancer groups with differing levels of immune infiltration. Specifically, we identified the SPP1 and EGF pathways as prominently active in the low immune infiltration group. SPP1, known for its role in modulating cytokine expression and immune checkpoint interactions, facilitates an immunosuppressive TME by influencing immune cell recruitment and activation. EGF acts through EGFR signaling to promote immune evasion, as evidenced by its association with poor responses to PD-1 inhibition therapies [124]. Our findings provide valuable insights into the intricate networks of cell–cell communication within the TME, suggesting potential targets for novel immunotherapy strategies. As the understanding of ligand–receptor interactions evolves, advancements in relevant databases can further enhance our comprehension of these complex communication networks, thereby improving cancer treatment outcomes.

## Data Availability

TCGA breast cancer data are available at the Genomic Data Commons (GDC) Data Porter (https://portal.gdc.cancer.gov/). GSE22219 and GSE176078 are available in the Gene Expression Omnibus (GEO) database.

## Supplementary Materials

The following supporting information can be downloaded at https://www.mdpi.com/article/10.3390/cells13181518/s1, Figure S1: Comparison of various clinical factors among the three immune infiltration groups; Figure S2: Tumor immune infiltration groups and their corresponding survival rates of UK breast cancer patients; Figure S3: Summary of the main study procedure and findings; Figure S4: Mutations in PIK3CA were not significantly associated with breast cancer patient survival; Figure S5: Comparison of VEGF signaling pathway activity in cellular communication networks of immune active and immune suppressed groups; Table S1: Pearson correlation coefficients across infiltration levels of various immune cell types and activity levels of angiogenesis and APM in the tumor microenvironment.

## Abbreviations

The following abbreviations are used in this manuscript:

TME	Tumor microenviroment
TMB	Tumor mutation burden
Breast cancer	BC
