# Supplementary material for: Characterizing the Tumor Microenvironment and Its Prognostic Impact in Breast Cancer
Source: Cells. 2024 Sep 10;13(18):1518. doi: 10.3390/cells13181518 (PMC11429566; doi:10.3390/cells13181518)
Supplement: Supplementary file 1 [file cells-13-01518-s001.zip › Supp. Figures S1-S5.pdf]

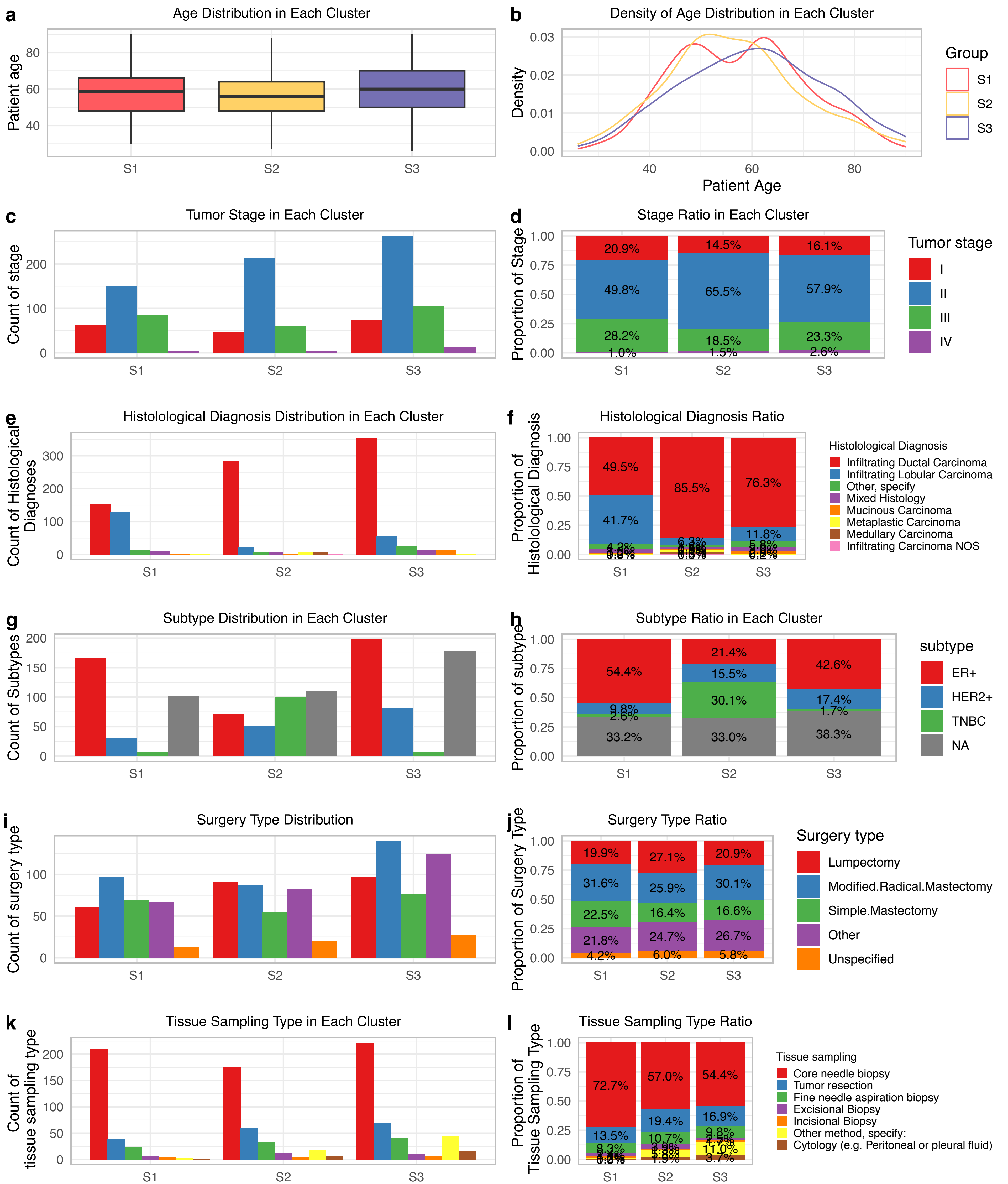

Supp. Figure1. Comparison of various clinical factors among the three immune infiltration groups.

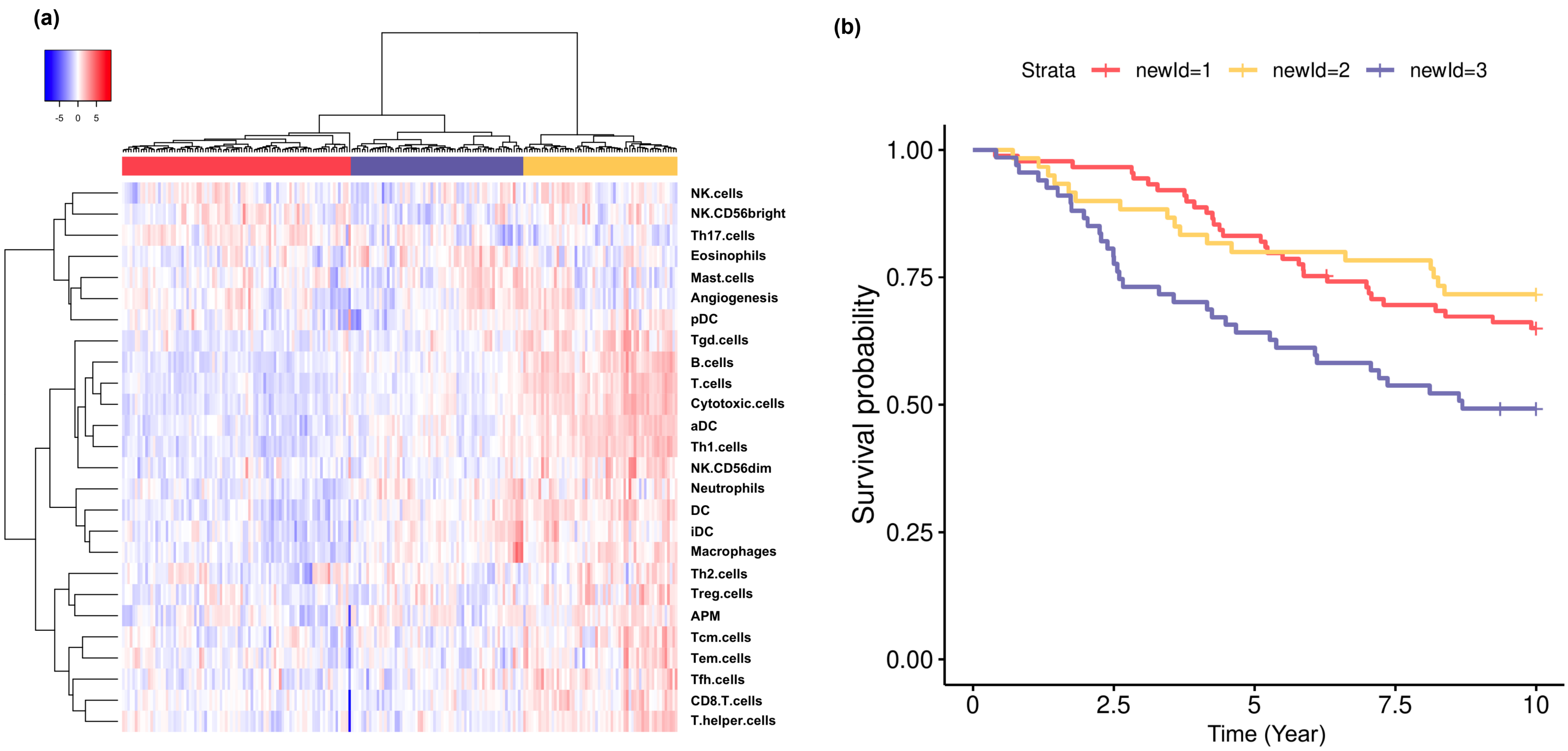

Supp. Figure2. Tumor immune infiltration groups and their corresponding survival rates of UK breast cancer patients.

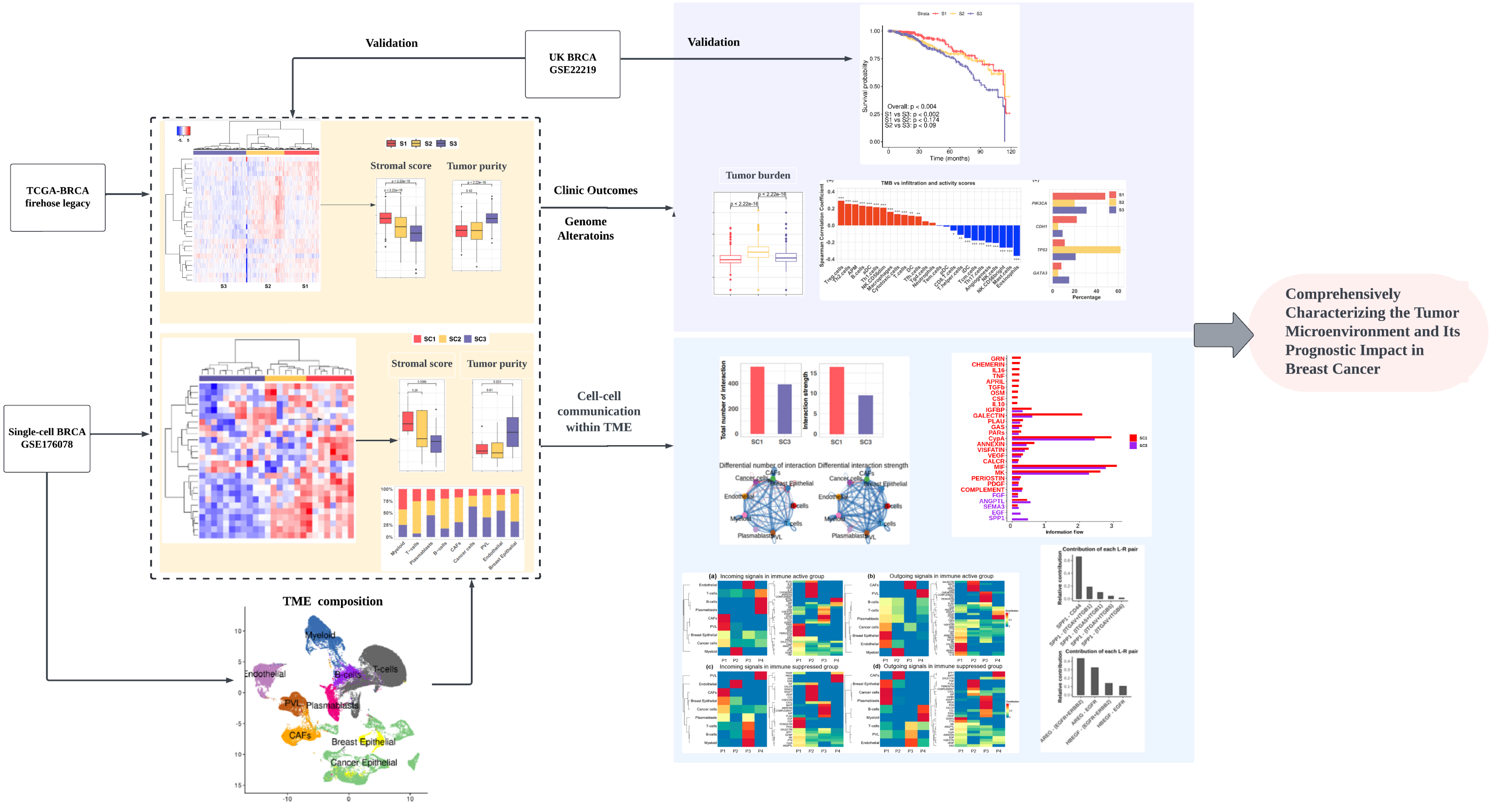

Supp. Figure 3. Summary of the main study procedure and findings.

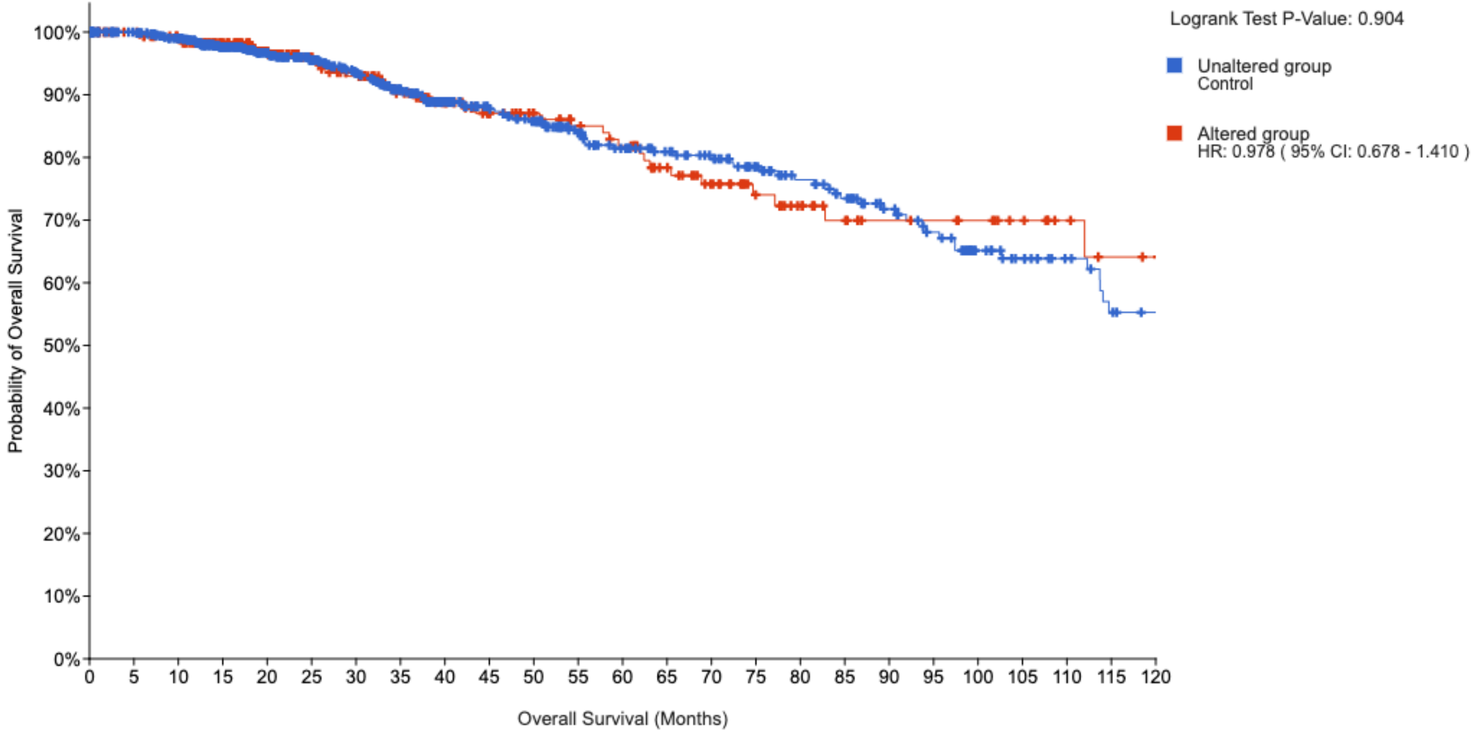

Number at risk (n)

Altered group	317	296	281	246	207	166	151	129	111	97	89	81	76	64	54	42	36	30	25	24	22	18	13	10	9
Unaltered group	659	616	582	496	430	377	338	289	251	223	209	175	158	146	136	119	106	96	81	71	55	47	39	32	29

Supp. Figure 4. The mutations of PIK3CA were not significantly associated with breast cancer patient survival in the TCGA dataset.

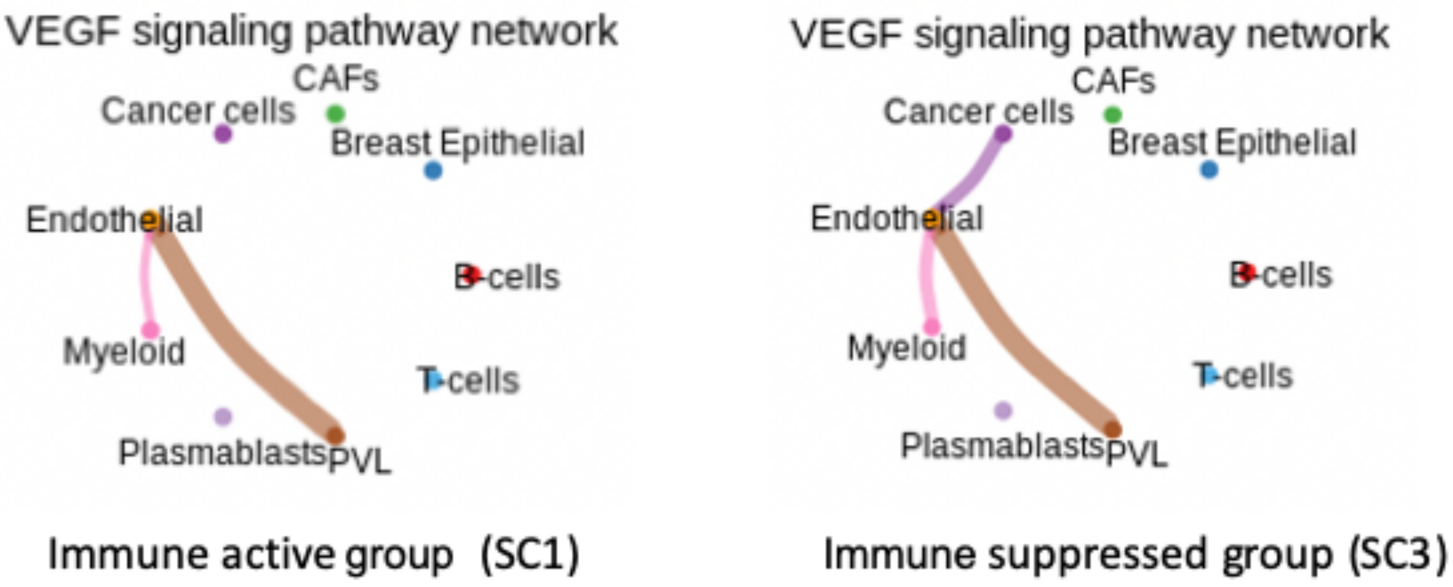

Supp. Figure5. Comparison of VEGF signaling pathway network in the immune active and immune suppressed BC patient groups.
